# High-Strength Double-Network Conductive Hydrogels Based on Polyvinyl Alcohol and Polymerizable Deep Eutectic Solvent

**DOI:** 10.3390/molecules28124690

**Published:** 2023-06-10

**Authors:** Yihan Zhang, Lei Jiang, Haibing Zhang, Qingyin Li, Ning Ma, Xinyue Zhang, Li Ma

**Affiliations:** 1 Qingdao Innovation and Development Center, Harbin Engineering University, Qingdao 266400, China; 2 State Key Laboratory for Marine Corrosion and Protection, Luoyang Ship Material Research Institute (LSMRI), Qingdao 266237, Chinaliqingyin@sunrui.net (Q.L.)

**Keywords:** conductive hydrogels, deep eutectic solvent, wearable sensors

## Abstract

Conductive hydrogels feature the flexibility of soft materials plus conductive properties providing functionality for effectively sticking to the epidermis and detecting human activity signals. Their stable electrical conductivity also effectively avoids the problem of uneven distribution of solid conductive fillers inside traditional conductive hydrogels. However, the simultaneous integration of high mechanical strength, stretchability, and transparency through a simple and green fabrication method remains a great challenge. Herein, a polymerizable deep eutectic solvent (PDES) composed of choline chloride and acrylic acid was added to a biocompatible PVA matrix. The double-network hydrogels were then simply prepared by thermal polymerization and one freeze-thaw method. The introduction of the PDES significantly improved the tensile properties (1.1 MPa), ionic conductivity (2.1 S/m), and optical transparency (90%) of the PVA hydrogels. When the gel sensor was fixed to human skin, real-time monitoring of a variety of human activities could be implemented with accuracy and durability. Such a simple preparation method performed by combining a deep eutectic solvent with traditional hydrogels offers a new avenue to construct multifunctional conductive hydrogel sensors with excellent performance.

## 1. Introduction

In recent years, a research boom has been set off by the emergence of soft and flexible conductors, such as flexible electronic skins [1,2], wearable sensor devices [3,4], human/machine interaction [5,6], supercapacitors [7,8], and actuators [9]. Small external stimuli, such as pressure [10,11], temperature [12,13], strain [14,15], etc., can be detected by these advanced conductors and converted into detectable and recordable electronic signals.

Conductive hydrogels, a soft material with a three-dimensional porous network structure, present high biocompatibility, tunable tissue-like mechanical properties, and minimal environmental destruction, all features which have attracted a myriad of attention in a wide range of areas such as artificial muscles [16,17], highly sensitive strain sensors [18], human–machine interfaces [19], and ionic skin [20].

Thus, considering the advantages of conductive hydrogels, various novel strategies have been adopted to improve the performance of conductive hydrogels to endow hydrogels-based sensors with remarkable properties. To data, the conventional strategy is to incorporate conductive active materials, such as conductive polymers [21,22], metal nanoparticles/nanowires [23,24], liquid metals [25,26], MXene [27,28], and carbon-based nanomaterials [29] into the hydrogels as an ideal flexible matrix. Nevertheless, the incompatibility between the conductive medium and the flexible substrates will cause it to form aggregates or uneven dispersion, which may compromise the mechanical properties, electrical conductivity, and transmittance of the conductive hydrogels. Considering this issue, it is greatly imperative to construct a transparent, high-strength conductive hydrogel whose conductive filler can be evenly dispersed in the matrix.

In recent years, ionic liquids (ILs) (a kind of low-melting salt) are usually called ‘green’ solvents [30,31]. With low volatility, thermal stability, and high ionic conductivity, they are widely used in the field of flexible electronics [32,33,34,35]. However, the development of ILs is often severely restricted due to its high toxicity and cost [36].

In view of the excellent performance and disadvantages of ILs, there is an urgent need for a new type of solvent. What is exciting is how deep eutectic solvents (DESs), as emerging alternatives to conventional ILs, have been cast with intensive research, and their emergence has brought forward a solution for the above problems. DESs, as a kind of low-melting eutectic mixture with a melting point that is lower than that of either of its components, can be simply prepared by mixing two or three inexpensive environmentally friendly raw materials in a certain range of molar ratio at a certain temperature [37,38,39]. In addition to the approximate properties of ILs (density, viscosity, refractive index, conductivity, surface tension, chemical inertness, etc.), DESs also have the characteristics of low cost, easy storage, simple preparation, and most of them are biodegradable [40].

The low melting point of DESs originates from the molecular complex itself formed between quaternary ammonium salts (hydrogen bond acceptors) and hydrogen bond donors. The molecular complex contains large asymmetric ions, which reduce the lattice energy of the system, resulting in a lower melting point [37]. The charge delocalization occurring through hydrogen bonding between for example a halide ion and the hydrogen-donor moiety is responsible for the decrease in the melting point of the mixture relative to the melting points of the individual components [37,39]. As compared to the conventional ILs, DESs, as a kind of sustainable solvent, have gradually attracted enough attention in many applications. In terms of flexible sensor equipment, DESs have the characteristics of biocompatibility [41], non-toxicity [42], and moderate ionic conductivity [43], providing researchers with a more optimized choice.

As a traditional hydrogel, polyvinyl alcohol (PVA) hydrogel has been widely used due to its good biocompatibility [44,45], non-toxicity [46,47], biodegradability [48], and stability [49]. Multiple cyclic freezing and thawing [50], as a common preparation method, is often used to pursue expected mechanical properties. It is a pity that this conventional method can moderately increase the crystallinity of the polymer chain while also reducing the transparency of the hydrogel. However, hydrogel structures constructed through non-covalent interaction bonding or double networks provide new inspiration for the design of hydrogels with efficient energy dissipation and excellent mechanical properties [51,52,53,54]. In this work, we introduced DES into PVA hydrogels by a single thermal polymerization and freeze-thaw after simple mixing to prepare composite hydrogels with excellent mechanical properties, transparency, and conductivity. Significantly, none of the chemical cross-linking agents were employed.

We chose a double-network conductive hydrogel with easy preparation, environmentally friendly, and low-cost features, which could be used for a wearable sensing device. The selected DES based on choline chloride (ChCl, hydrogen bond acceptor) and acrylic acid (AA, hydrogen bond donor), as a classic DES system with low cost and green environmental protection, provided a neutral ligand AA that can be polymerized by free radicals. While polymerizable deep eutectic solvent (PDES) and PVA formed an interpenetrating cross-linked network, there were also abundant hydrogen bonding sites between them, which would effectively enhance the mechanical strength of pure PVA hydrogels. The formation of an interpenetrating network with PVA after DES polymerization could solve the leakage problem of traditional ionic gels. High density hydrogen bond interaction and an interpenetrating polymer network promoted DES to disperse stably in the system. In addition, PDES imparted ionic conductivity to the hydrogels through the ions ionized by choline chloride, which demonstrated the potential of these applications in wearable sensing devices.

## 2. Results and Discussion

**Figure 1:**
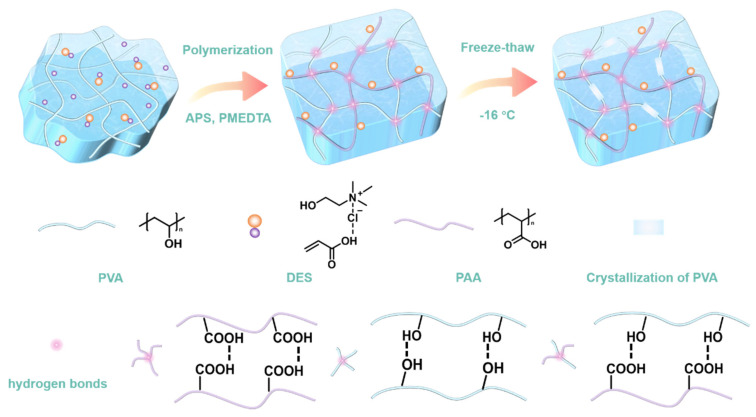
Schematic for the preparation and the network structure of PVA-DES hydrogels.

In order to enhance the mechanical strength and conductivity of hydrogels, the preparation of PVA-DES hydrogels requires the construction of dual networks, namely PVA network and PDES network. As shown in Figure 1, a uniform dispersion could be obtained by mixing the newly prepared PVA solution with the DES (ChCl/AA) in a certain proportion. After the initiator APS and the accelerator PMEDTA were evenly dispersed in the mixture, the AA monomer in the DES was polymerized by thermal polymerization to form a second network on the original PVA network. Subsequently, PVA-DES hydrogels possessing excellent mechanical strength could be obtained by one rapid freeze-thaw cycle, during which the phase separation into PVA-rich and water-rich phases results in the repulsion of a PVA polymer chain to form aggregated regions of PVA [55]. As the PVA chains came into close contact with each other, crystallite formation and hydrogen bonding occurred [55]. In addition to the hydrogen bond between -COOH groups in PDES, -COOH groups could also form hydrogen bonds with hydroxyl groups in the PVA. Choline chloride in PDES, which could ionize positive/negative ions in an external electrical field, assumed the role of ionic conductivity [56]. While PAA, as a flexible macromolecular chain, played the role of strengthening and toughening as a second network.

Figure S1a was the schematic diagram and reaction formula of the DES synthesis, from which it could be clearly seen that the DES was colorless, homogeneous, and transparent. From the infrared spectra of the DES and its individual components (Figure S1b), the carboxylic acid band at 1717 cm^−1^ exhibited that AA presented the characteristics of a hydrogen-bonded body [57]. Furthermore, the melting point of the synthesized DES was about −5 °C, which was much lower than that of AA (~14 °C) and ChCl (~302 °C) [58] (Figure S1c).

**Figure 2:**
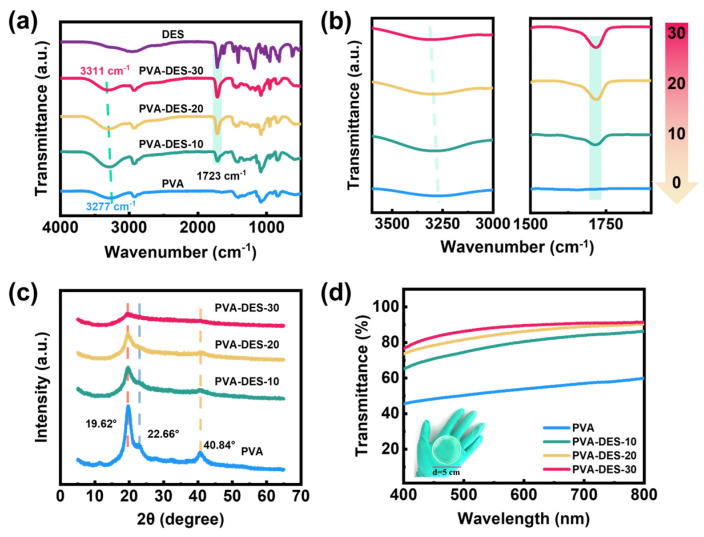
Characterization of PVA-DES-X gels. (**a**) ATR-FTIR spectra for PVA-DES-X (X = 0, 10, 20, 30) gels and DES (ChCl/AA). (**b**) Partial enlarged view of reflected infrared spectrum for the PVA-DES-X gels. (**c**) XRD patterns for the pure PVA powder and PVA-DES-X gels. (**d**) UV-vis spectra of all hydrogels; the inset is the digital photo of PVA-DES-30 gel.

The broad absorption bands around 3277 cm^−1^ in the ATR-FTIR spectrum of the PVA hydrogel were attributed to the free -OH groups and the -OH groups forming hydrogen bonds with each other [59]. After the addition of the DES, the bank shifted to 3311 cm^−1^, indicating that part of the -OH groups form hydrogen with the -COOH groups [59,60,61]. The peak at 2929 cm^−1^ was the stretching vibration peak of C-H. The C=O belonging to 1717 cm^−1^ in the DES also appeared in the hydrogels, which confirmed the successful introduction of the DES (Figure 2a). As shown in Figure 2b, with the addition of DES, the stretching vibration peak of the hydroxyl group gradually shifted to the high field, the peak width of the characteristic peak of the carbonyl group gradually increased, which was caused by the formation of a hydrogen bond between –OH groups on the PVA chain and the -COOH groups of the PDES. X-ray diffraction (XRD) was used to study the effect of DES on the crystallinity of the PVA (Figure 2c). It could be seen that, when DES was added, the strength of the PVA crystallization peaks [corresponding to 19.6°, 22.6°, and 40.84° to the lattice planes of (1 0 1), (2 0 0), and (1 0 2)], respectively [62]), decreased significantly, because the -COOH groups in the acrylic acid formed hydrogen bonds with the -OH groups in the PVA, which inhibited the crystallization of the PVA chain [18]. Some similar phenomena have been reported in the past [63,64]. The transmittance of hydrogels measured by the Ultraviolet-Visible spectrum (UV-Vis), and the digital photos of the physical map, could confirm this view. The transparency of samples in the visible range was gradually improved when the content of DES was increased (Figure 2d). Compared with pure PVA hydrogel, the transmittance of the PVA-DES-30 gel reached 90% in the visible light region (400–800 nm). The PVA-DES-30 gel in the inset exhibited high transparency. The white opaque area formed by the microcrystalline region of the original PVA gradually disappeared with the introduction of the DES (Figure S2). As a semi-crystalline polymer, PVA is subject to phase separation during the freeze-thaw cycle due to the growth of ice crystals, which allows the PVA phase to be concentrated and thus favors chain entanglement. The hydroxyl groups on the PVA chains form strong hydrogen bonds with each other, resulting in the formation of microcrystals, which explains the strongest diffraction peaks and the lowest transparency of pure PVA after a single freeze-thaw in Figure 2c,d. In contrast, the DES as a small molecule can easily free itself around the PVA chain and form hydrogen bonds with the PVA chain. After thermal polymerization, the formation of PDES molecular chains prevents PVA molecular chains from entangling with each other. When the ice crystals grow, PDES and PVA are compressed together, but the stronger hydrogen bonding between -COOH and -OH inhibits the PVA chains from forming intramolecular or intermolecular hydrogen bonds themselves (Figure S3).

**Figure 3:**
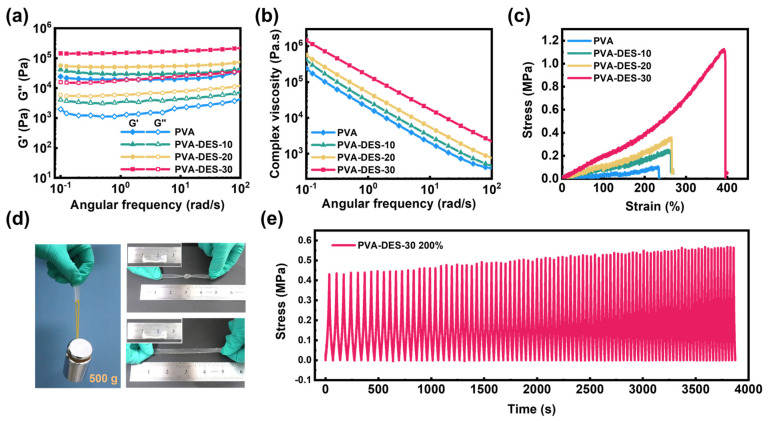
Rheological and mechanical performance of PVA-DES-X gels. (**a**,**b**) The frequency dependence of G′, G″, and complex viscosity for hydrogels with different DES content at 1% strain on the angular frequency range from 0.01 to 100 rad s^–1^, respectively. (**c**) Tensile stress-strain curves of PVA-DES-X gels. (**d**) The displaying of mechanical properties for the PVA-DES-30 gel, including lifting a weight of 500 g, stretching, and knotting. (**e**) The cyclic tensile test for nealy 100 times cycles for the mechanical behavior of the PVA-DES-30 gel under a strain of 200%.

Rheological testing of hydrogel materials is essential for evaluating their viscoelastic behavior. Figure 3a shows the frequency dependence of storage modulus (G′) and loss modulus (G″) of hydrogels under constant strain (1%) and temperature (25 °C). The G′ of the hydrogels was consistently higher than its G″, indicating dominant elastic state behavior over the entire frequency range [65]. Higher G′ was obtained with an increase in DES content. The G′ of the hydrogel increased slightly with an increase in frequency. However, the apparent increase in G″ with increasing frequency might be caused by more and more hydrogen bond loss at high frequencies. The complex viscosity of all samples had a consistent downward trend with an increase in frequency (Figure 3b), which was caused by the shear thinning characteristics of the hydrogel materials [62]. Furthermore, the gel point only occurred in the pure PVA gels at 0.25 °C (Figure S4). In contrast, in the gel samples containing PDES, there was a sudden modulus change point in the range of −4 °C to −6 °C, with a precipitous drop in both G′ and G″, but the former was still higher than the latter, proving that these hydrogels remain in a solid state. As the temperature continued to rise to 100 °C, the G′ was much higher than the G″, demonstrating that the PVA-DES-X (10–30) exhibited a highly elastic state. To further investigate the effect of DES on the mechanical properties of the materials, a stress-strain curve was obtained through an uniaxial tensile test (Figure 3c). The increase in DES contents from 0–30% exhibited a continuous improvement in mechanical properties. The tensile strength and elongation at break increased from 0.10 MPa to 1.1 MPa and from 235% to 390%, respectively. In addition, Young’s modulus and toughness increased significantly with the increase in DES content (Figure S5a). When the DES content was 30%, Young’s modulus and toughness reached 0.19 MPa and 2.4 MJ/m^3^, respectively, which were 1.9 and 12 times higher than those of the pure PVA gel. The rigid network formed by PDES and PVA could effectively enhance the mechanical strength of the hydrogel. After analysis, there are several reasons that may explain the enhanced mechanical properties. Firstly, the increased polymer content would be beneficial for improving the mechanical properties of hydrogels (Figure S6). In the hydrogel samples of pure PVA and PVA-DES-10 gels, the water content of both remained high and close after 12 h of confined thermal polymerization. This is due to the fact that the DES content only occupies 10% of the total gel mass, so it has little effect on the water content. The introduction of PDES also reduced the mass fraction of PVA within the gels, which is consistent with the similar tensile test performance of PVA and PVA-DES-10 gels. As the DES content continued to increase to 20wt%, the water content showed a significant decrease, and the mechanical properties of the PVA-DES-20 hydrogel became stronger. However, when the DES mass fraction was increased to 30wt%, the mechanical properties of the hydrogel were significantly improved. This was probably due to the enhanced density of the PDES network, which allowed the formation of a dense interpenetrating network with the PVA network, as well as the decrease in water content to only 56%, resulting in the most outstanding mechanical properties of the PVA-DES-30 gel. In addition, the covalent dual network of the PDES and PVA chains together exhibits higher strength and toughness compared to a single PVA network. The toughness is attributed to the internal fracture of the covalent bonds in the fragile first network, which dissipates energy and increases the resistance to crack extension [66]. Finally, weak hydrogen bonds can be built as sacrificial forces to dissipate fracture energy under external forces, resulting in higher toughness for the materials. Due to its solid mechanical properties, the PVA-DES-30 gel was undamaged in the process of large deformation such as stretching and knotting, and it could also undertake a weight of 500 g (Figure 3d). As shown in Figure S5b, it could also be stretched to 400% of its original length. In addition, to investigate the stability of hydrogels, tensile cycle tests without any resting time were carried out (Figure 3e). The PVA-DES-30 gel remained stable under 100% strain tension for nearly 100 times cycles, which confirmed the stability of the network structure. After many cycles, the tensile stress increased slightly due to the loss of water from the hydrogel into the air. The introduction of the DES not only provided for the formation of a double interpenetrating network system, but also increased the content of the hydrogen bonds, which improved the mechanical strength of the hydrogels. The 3D network structure of the hydrogels also has a great impact on their mechanical properties. Concerning the cross-sectional morphology of the PVA-DES gel, the original PVA gel had a rough cross section without porous structure (Figure S7a). However, when DES was introduced into the system, the gel showed a three-dimensional porous network structure (Figure S7b). Such a uniform and dense microporous structure is conducive to protecting the pore from collapse, thereby improving the stability of the network structure and providing more space for the free ions in the channel [18].

**Figure 4:**
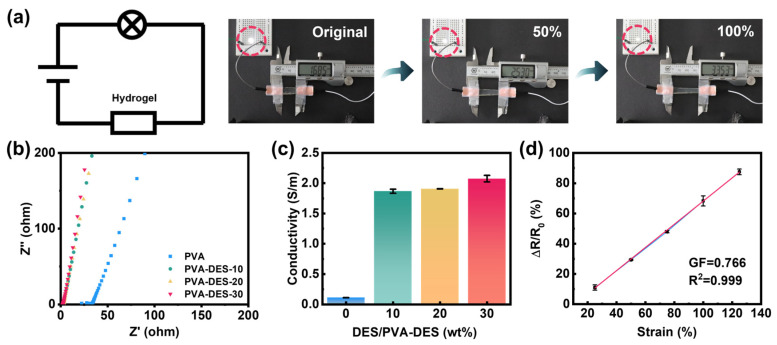
Ion-conductive and strain-sensing properties of PVA-DES-X hydrogels. (**a**) Photograph of the different stretch lengths of PVA-DES-30 gel (original length, 50% strain, 100% strain) and LED light circuit in series. (**b**) Electrochemical impedance spectroscopy (EIS) plots of PVA-DES-X gels. (**c**) Plots of calculated conductivity values for PVA-DES-X hydrogels. (**d**) Relative resistance changes rate of hydrogel-based strain sensor as a function of strain (25–125%).

The presence of choline in the PDES is the source of the conductivity of the hydrogels. In the circuit, choline chloride will produce free-moving positive/negative ions, which are limited by the formed polymer network structure. Therefore, its conductivity will also change with the change in network structure. As shown in Figure 4a, when the gel and the LED lamp were connected in series at a constant voltage of 5 V, it was intuitively observed that when the gel was stretched from its original length to 100% strain, the brightness of the LGD also changed. As the gel gradually changed from its original state to 50% and 100% strain, the brightness of the LGD decreased gradually, which presented the influence of the deformation of the hydrogel on its internal resistance. When conductors are stretched, they tend to shrink in the transverse direction of the stretch, which leads to an increase in conductor resistance according to the formula R = ρL/A, where ρ is the electrical resistivity, L is the length, and A is the cross-sectional area of the conductor (Figure S8).

The proportions of PDES as ionic conductors will affect the conductivity of PVA-DES gels in the absence of other external conductors. Therefore, electrochemical impedance spectroscopy (EIS) was used to measure the conductivity of gels with ion movement as the conductivity mechanism (Figure 4b,c). During the EIS test, it was necessary to clamp the thin hydrogels between two copper electrodes for the test (Figure S9). By extrapolating and fitting the imaginary part of impedance (Z″), the ohmic conductivity can be obtained [26]. With the increase in the mass ratios of DES, the conductivity of samples increased from 0.11 S m^−1^ (pure PVA gel) to 2.11 S m^−1^ (PVA-DES-30 gel). To explore the responsiveness and sensitivity of the PVA-DES-30 gel as a strain sensor, the relative resistivity change rates of the gel under different strains were investigated (Figure 4d). For strain sensors, sensitivity and accuracy are two important parameters, which are closely related to the gauge factor (GF) and the linearity (R^2^) obtained by fitting the curve, respectively [67]. GF could be obtained from the slope of the relative resistance change versus the strain curve [68,69]. The GF and R^2^ of the strain sensor reached 0.766 and 0.999, respectively.

The relative resistivity change rates all exhibited linear changes over the strain range from 25% to 125%, which indicates the wide working range and precise sensitivity of the PVA-DES-30 gel. The gel could still maintain a fairly stable responsiveness under 100 cycles of 125% strain, demonstrating its stability and durability as a strain sensor (Figure S10). As shown in Figure S11, the response time and recovery time of the sensor under 25% strain tension were 500 ms and 800 ms, respectively. Considering the excellent mechanical properties, transparency, ionic conductivity, and stable sensitivity, the PVA-DES-30 gel can become a promising candidate in the field of strain sensing.

**Figure 5:**
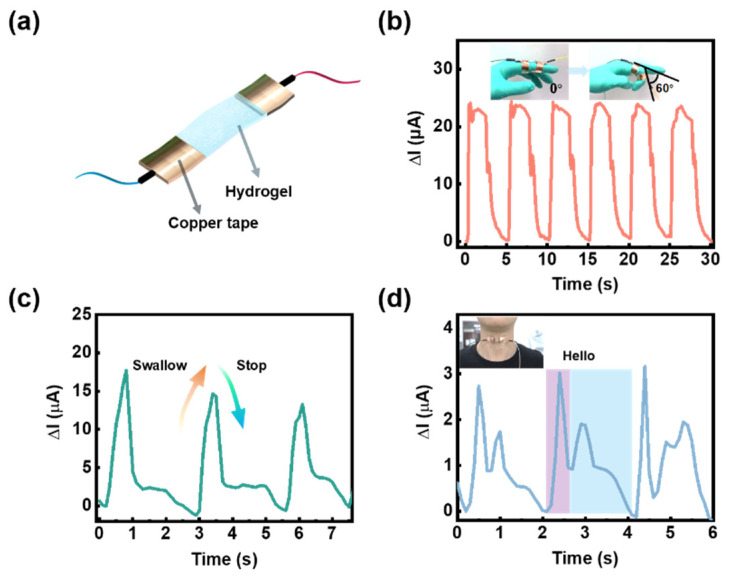
Performance testing of the PVA-DES-30 gel as a wearable sensor. (**a**) The schematic diagram of the PVA-DES-30 gel was fabricated as a wearable sensor; the gel was fixed with copper tapes attached to both sides and was connected to the electrochemical workstation by connecting wires. (**b**) The sensor curve of the detection of the degree of bending of the finger by the PVA-DES-30 gel; the illustration shows the operation picture of the finger bending sensor. (**c**) Swallowing induction curve by sticking the gel to the throat. (**d**) Sound sensing of the word ‘hello’ by the gel; the inset is a diagram of the device under test.

After application as a strain sensor has been verified, the PVA-DES-30 gel could be expected to be used as a human body wearable sensing device to monitor human activity in real time. The wearable sensor functions by fixing the hydrogel conductor to the skin with copper conductive tape welded with wires (Figure 5a). The corresponding real-time current changes appeared on the electrochemical workstation, accompanied by repeated flexion and extension of the fingers at an angle of 0° to 60° without delay (Figure 5b). In addition, when the fingers were kept at different degrees of bending (0°, 30°, 60°) for 5 s, the output current signal could still remain stable (Figure S12a). When it was fixed to the wrist, it could exhibit obvious and regular current changes as the fist was clenched and relaxed (Figure S12b). Compared with the monitoring of limb movement, the monitoring function of human swallowing and language might become particularly important for wearable sensors in the medical field, especially for the elderly. As shown in Figure 5c,d, after fixing the gel to the throat, the swallowing and speaking behaviors of the human body could be detected through a regular current signal. Even the two syllables of the words spoken by the volunteers were accurately distinguished. More importantly, the gel sensor could accurately output the characteristic signal of the weak strain, which reflected its solid stability. These weak and complex changes in the human body could be accurately distinguished by the changes in the current signal, which confirmed the potential of the PVA-DES gels for detecting real-time human motions.

## 3. Experimental Section

### 3.1. Materials

Poly(vinyl alcohol) (PVA 1799, degree of hydrolysis: 99%, average molecular weight: 77,000) was purchased from Sinopharm Chemical Reagent Co., Ltd. (Shanghai, China). Acrylic acid (AA, >98%) and N,N,N′,N′,N″-Pentamethyldiethylenetriamine (PMDETA, 98%) were provided by Shanghai Macklin Biochemical Co., Ltd. (Shanghai, China). Choline chloride (ChCl, 99%) was supplied by Innochem (Beijing, China) Technology Co., Ltd. (Beijing, China). Ammonium persulphate (APS) was bought from Fuchen (Tianjin, China) Chemical Reagent Co., Ltd. (Tianjin, China). The deionized water (DI) was purified by a Milli-Q purifier. All reagents were used as received without any further purification.

### 3.2. Preparation of Hydrogels

#### 3.2.1. Preparation of DES (ChCl/AA)

Deep eutectic solvent composed of ChCl and AA was synthesized according to the method provided in the literature [57]. In the initial process, ChCl was dried in a vacuum oven at 60 °C for 2 h, and AA was dried with 4 Å molecular sieves. ChCl and AA (molar ratio of 1:2) were then added to a round-bottom flask and stirred for 2.5 h at 90 °C to obtain a homogeneous and transparent DES. Subsequently, the prepared DES was immediately transferred into a vacuum desiccator with silica gel.

#### 3.2.2. Preparation of PVA-DES Hydrogels

As shown in Table 1, PVA-DES hydrogels composed of different amounts of DES were labeled as PVA-DES-X, where X represented the mass fraction of DES in hydrogels. After PVA powder was added to deionized water, it was quickly stirred at 95 °C for 1.5 h to obtain 12.5 wt% PVA solution. DES was added to the newly prepared PVA solution, stirred at 80 °C for 1.5 h, and then slowly cooled to room temperature to obtain a colorless and uniformly dispersed mixed solution. The initiator APS (its mass is 2% of the mass fraction of acrylic monomer) and the accelerator PMEDTA were added to the above solution. In order to make the initiator and accelerator evenly dispersed in the system, the mixture needed to be stirred at room temperature for 1 h. To complete the free radical polymerization process, the mixture needed to be degassed in advance and transferred to a closed Petri dish. The polymerization process proceeded at 60 °C for 12 h. After the culture dish was cooled to room temperature, it was then transferred to a −45 °C cold well and frozen for 2 h. As a blank control, the pure PVA hydrogel was also prepared by the same method.

### 3.3. Construction of Strain Sensor and Its Integration as a Wearable Sensor

The prepared PVA-DES-30 gel was cut into long strips of 2.0 cm × 0.5 cm (length × width), the ends of which were sealed with copper tape connecting wires to obtain strain sensors. The prepared strain sensors were fixed onto human finger joints, throat, wrist, and other parts to detect human activities, such as finger bending, swallowing, speaking, and other signals.

### 3.4. Characterization

Fourier transform infrared (FTIR) spectra of samples were obtained from a Nicolet 6700 instrument with attenuated total reflection (ATR) in the wavenumber range of 400–4000 cm^−1^. Wide angle X-ray diffraction (XRD) spectra of hydrogels were recorded by the Rigaku TTR-Ⅲ X-ray diffractometer (Japan) at a scanning rate of 5° min^−1^ over the 2θ range of 5–65°. Samples needed to be freeze-dried and dehydrated before testing. The morphologies of the prepared hydrogels were characterized by scanning electron microscopy (JSM-IT300LV) with a resolution of 3 mm and a voltage of 5 KV. Samples were first quenched in liquid nitrogen and then freeze-dried. The rheological properties of the hydrogels were demonstrated by the TA DHR-2 rheometer. In the oscillation mode, the sample is tightened by a 25 mm stainless steel parallel plate and sealed by paraffin liquid to avoid water loss. UV–vis spectrum characterization of the hydrogels was carried out on a SP-1702 spectrophotometer in the wavelength range from 400 to 800 nm with a resolution of 1 nm. Differential scanning calorimetry (DSC) analyses were conducted on a DSC2A00724 instrument. The specimens were heated from 60 °C to 120 °C at a rate of 10 °C min^−1^. Tensile testing of hydrogels was performed using an Instron 3365 universal mechanical tester at a speed of 20 mm min^−1^. The prepared hydrogels were cut into uniform dumbbell shaped samples (length 25 mm, width 4 mm, and thickness 2 mm). Young’s modulus and toughness could be assessed by the linear slope of the stress-strain curve and the area enclosed by the curve, respectively [29]. Electrochemical impedance spectroscopy (EIS) plots of hydrogels were measured by a PARSTAT 2273 electrochemical comprehensive tester. The hydrogels were sheared into 11 mm diameter shapes and sandwiched between two gaskets. The frequency range was set from 0.1 Hz to 10^6^ Hz, and each sample was tested three times on average. The ionic conductivity of the hydrogels could be calculated accordingly as follows:.

$$\sigma =L∕\left(R_{b}A\right)$$

where σ, L, R_b_, and A are ionic conductivity (S/m), thickness (m), resistance (Ω), and cross-sectional area (m^2^) of the hydrogels, respectively [56].

The resistance changes of the hydrogels were measured by the CHI660E workstation. The relative change in resistance was calculated by the following formula.

$$\Delta R∕R_{0}=(R-R_{0})/R_{0}$$

R and $R_{0}$ are the original and deformed resistance values of the hydrogel, respectively [70].

The water content of these hydrogel samples was determined by the percentage change in weight in the water-saturated state (W_0_) and in the dry water-loss state (W_d_). The formulae are as follows:

$$water\;content\left(wt\%\right)=[(W_{0}-W_{d})/W_{0}]\;*\;100$$

The surface water of the freshly prepared hydrogel was wiped with paper and the weight at this point was recorded as W_0_, after which it was placed in an oven at 120 °C to dry the water and the mass at this point was recorded as W_d_.

## 4. Conclusions

In summary, we have successfully synthesized the PVA-DES hydrogel with high tensile strength, high transmittance, and conductivity by a simple and clean method of thermal copolymerization followed by a freeze-thaw cycle. Higher tensile properties (1.1 MPa) were obtained due to the interpenetrating network formed by the interpenetrating polymer networks of PDES and the original PVA chains, and the stronger hydrogen bond interactions. The light transmittance (90%) of the gel had also been greatly improved due to the inhibitory effect of PDEs on PVA chain crystallization. The introduction of PDES endowed PVA hydrogel with excellent conductivity (1.9 S m^−1^). Under a certain voltage, the anions and cations provided by choline chloride could move continuously in the hydrogel network. With the excellent conductivity, high strength, and stable responsiveness to various strains, the PVA-DES hydrogels have better development potential in the field of wearable devices.

## Figures and Tables

**Table 1:** Formula of different hydrogels in the work.

Hydrogels	PVA (g)	DI (g)	DES (g)	APS (g)	PMDETA (µL)	Content of DI (wt%)
PVA	1.5	10.5	0	0	0	87.5
PVA-DES-10	1.5	10.5	1.33	0.014	5.6	78.7
PVA-DES-20	1.5	10.5	3.00	0.031	12.4	69.8
PVA-DES-30	1.5	10.5	5.14	0.052	21.2	61.3

## Data Availability

Data is contained within the article or supplementary material. The data presented in this study are available in supporting information.

## Supplementary Materials

The following supporting information can be downloaded at: https://www.mdpi.com/article/10.3390/molecules28124690/s1, Figures S1–S12.
