# Supplementary material for: High-Strength Double-Network Conductive Hydrogels Based on Polyvinyl Alcohol and Polymerizable Deep Eutectic Solvent
Source: Molecules. 2023 Jun 10;28(12):4690. doi: 10.3390/molecules28124690 (PMC10301373; doi:10.3390/molecules28124690)
Supplement: Supplementary file 1 [file molecules-28-04690-s001.zip › molecules-2427121-supplementary.pdf]

# Supporting information

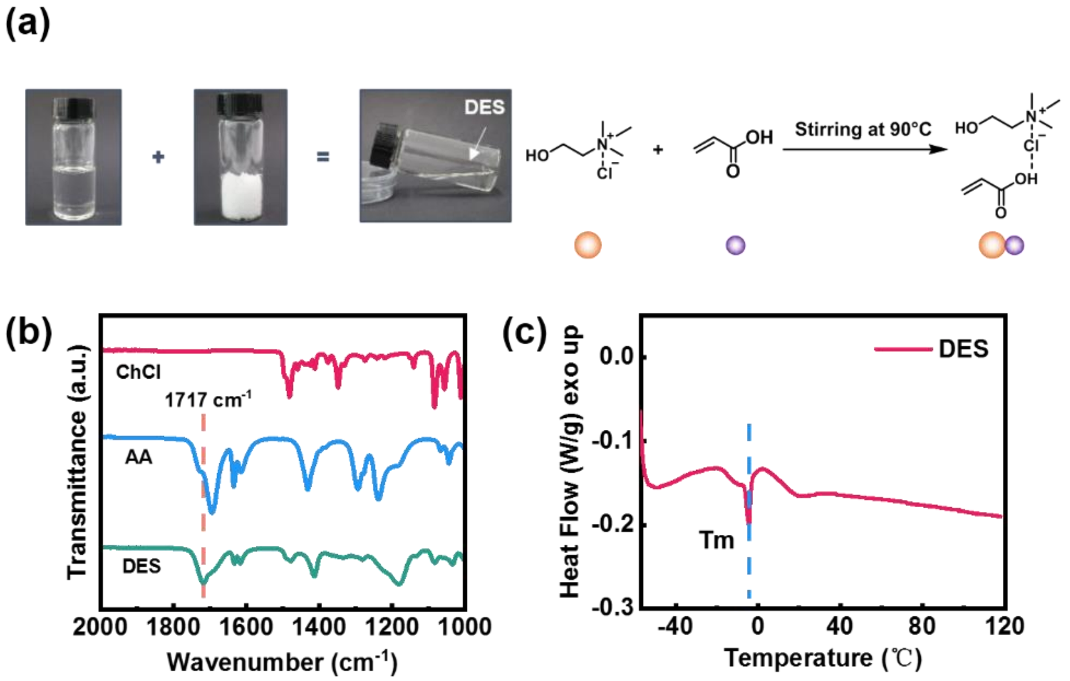


Figure S1. Synthetic route and characterization of DES. (a) The raw materials for the preparation of DES including choline chloride acrylic acid, which were mixed and stirred at 90°C for a period of time to obtain a colorless and transparent liquid-DES. (b) ATR-FTIR spectra of the DES, AA and Choline Chloride. (c) DSC curve of the DES. The test was carried out under a nitrogen atmosphere with a heating rate of 10°C $min^{-1}$.

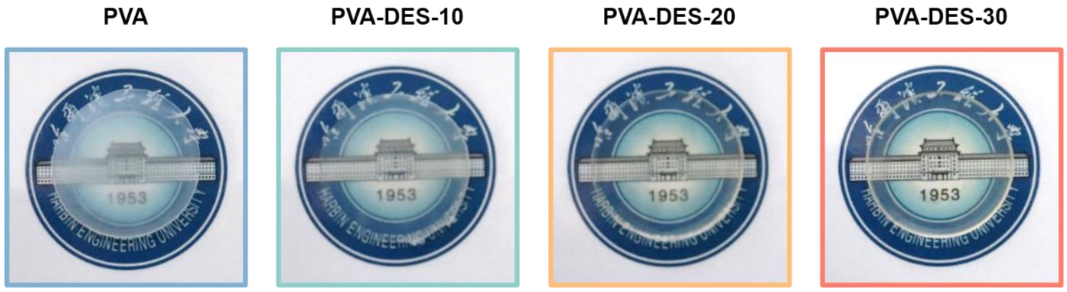


Figure S2. Digital photos of PVA-DES gels containing different DES mass fractions.

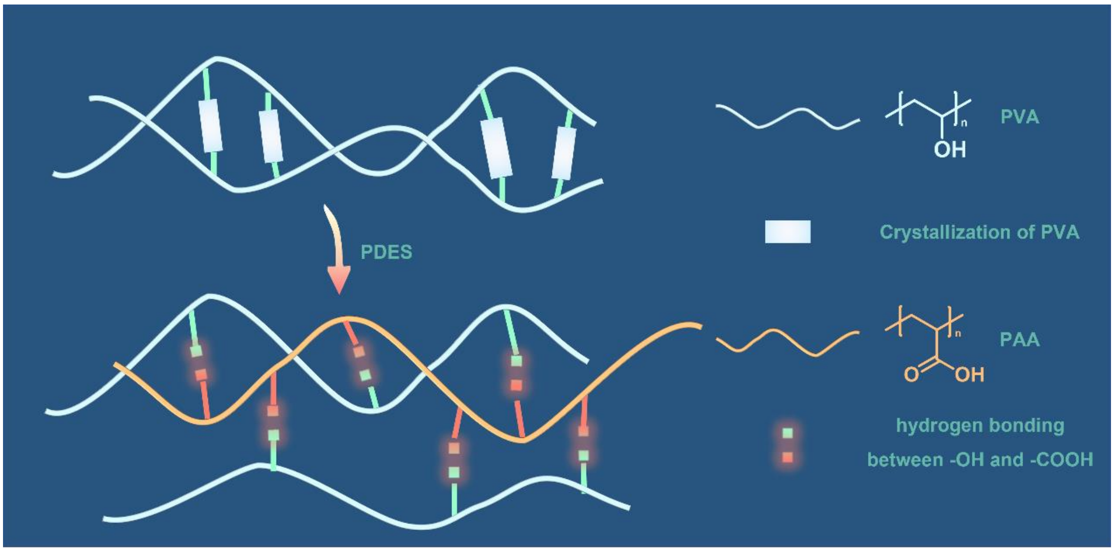


Figure S3. Schematic representation of the mechanism by which PVA crystallisation is inhibited. For the pure PVA hydrogel, during the freezing process, the PVA molecular chains are compressed into the interstices of the ice crystals as they grow, which makes the PVA molecular chains entangled and tight. When PDES is introduced into PVA, the PDES molecular chains prevent the chain entanglement of PVA molecular chains, reducing the chance of PVA forming intermolecular hydrogen bonds. In addition, the PAA chain in PDES contains a large amount of -COOH, which will form stronger hydrogen bonding with the hydroxyl groups in the PVA chain, thus inhibiting the formation of microcrystals.

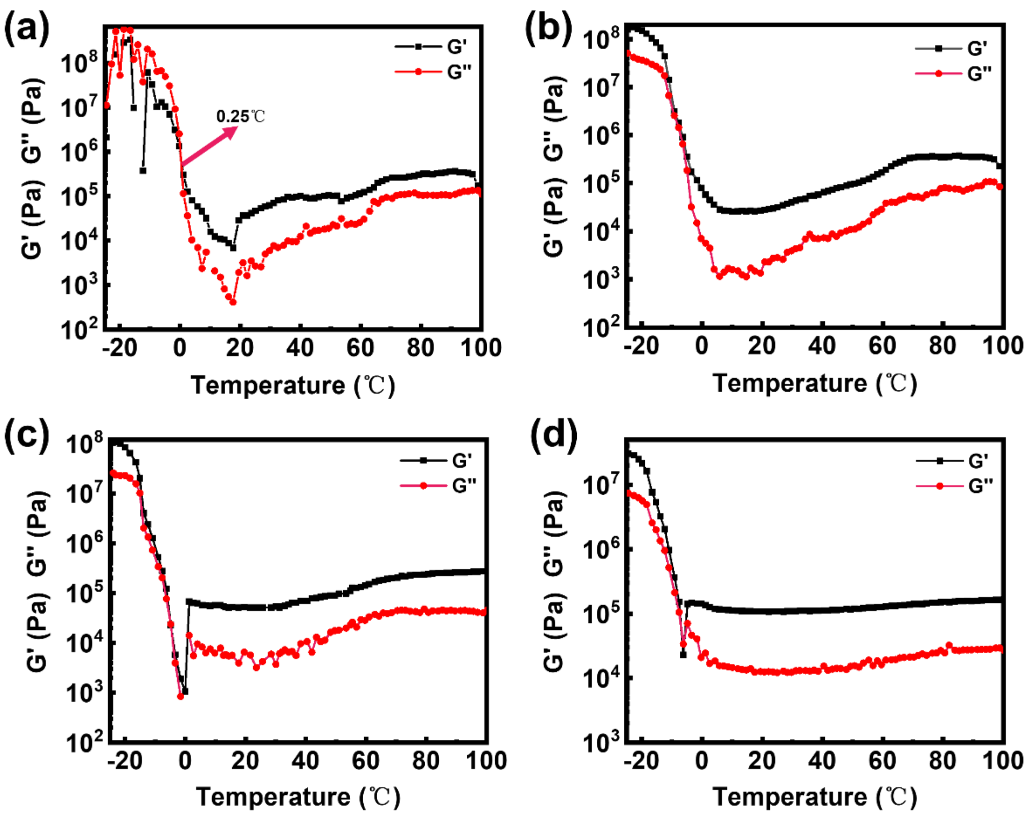


Figure S4. Rheological performance of PVA-DES-X gels. (a-d) The temperature dependence of G' and G" for PVA-DES-X gels (X=0,10,20,30) content at 1% strain on the angular frequency of 1 rad/s, respectively.

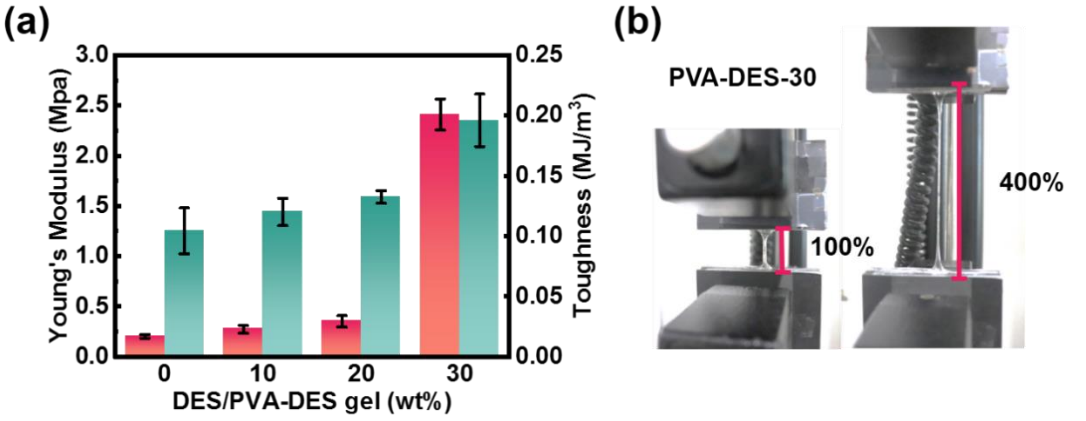


Figure S5. Mechanical properties of PVA-DES gels. (a) Young's modulus and toughness of PVA-DES gels with increasing DES mass fractions. (b) Digital photos of the pristine state of PVA-DES-30 gel and stretched to 400% strain.

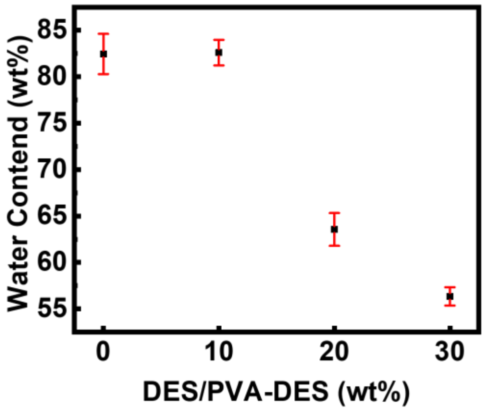

Figure S6. Water content (wt%) of PVA-DES-X hydrogels.

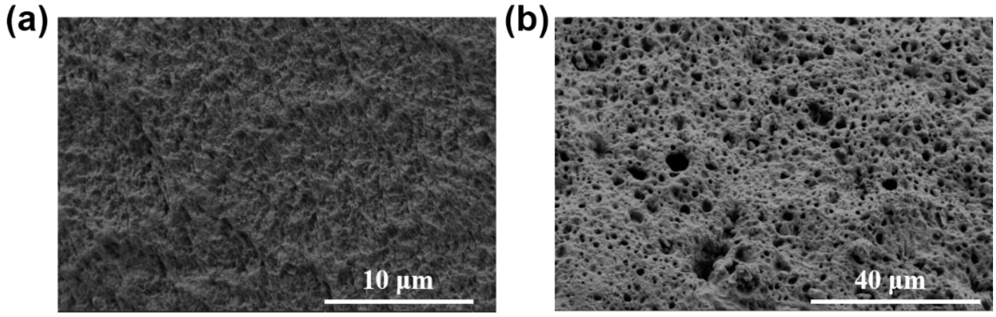

Figure S7. Scanning electron microscopy (SEM) images of the cross section for PVA gel (a) and PVA-DES gel (b).

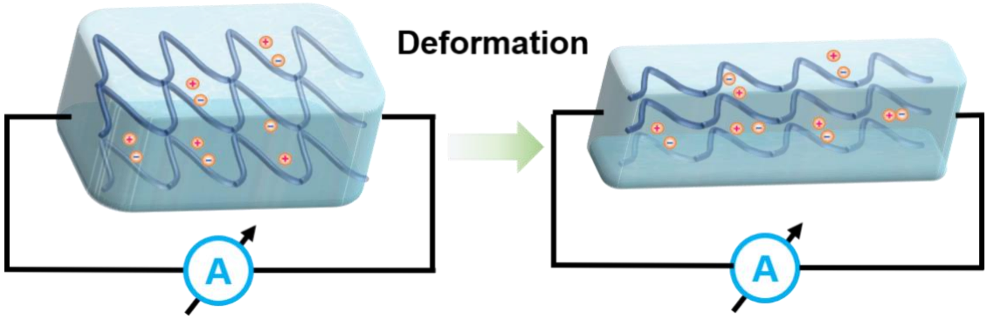

Figure S8. Schematic diagram of the strain response of the hydrogels, showing the strain sensing mechanism.

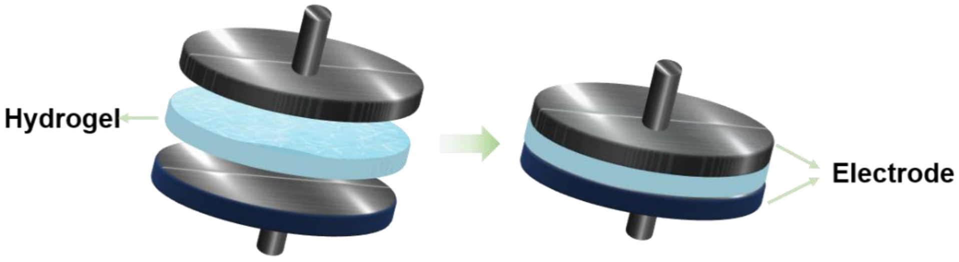

Figure S9. Schematic representation of the hydrogel that was cut into a thin film being tightly fitted between

electrode sheets to obtain electrochemical impedance spectroscopy (EIS) plots.

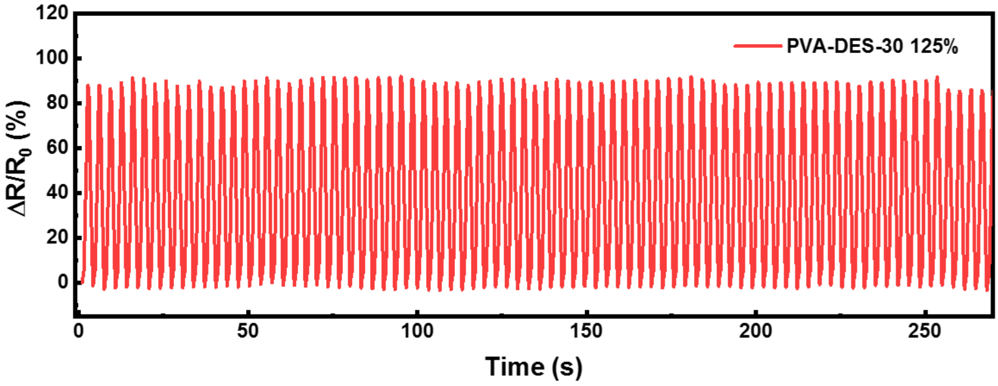


Figure S10. Cycling testing of sensing performance of PVA-DES-30 gel at 125% strain.

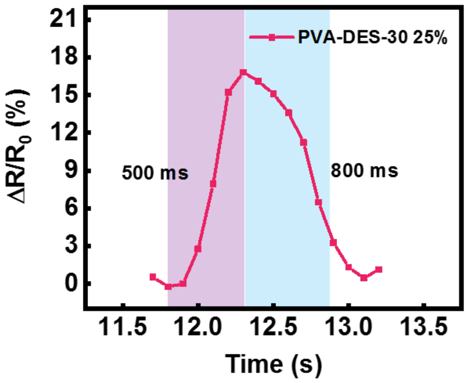


Figure S11. Response time and recovery time of PVA-DES-30 strain sensor under tension cycle of 25% strain.

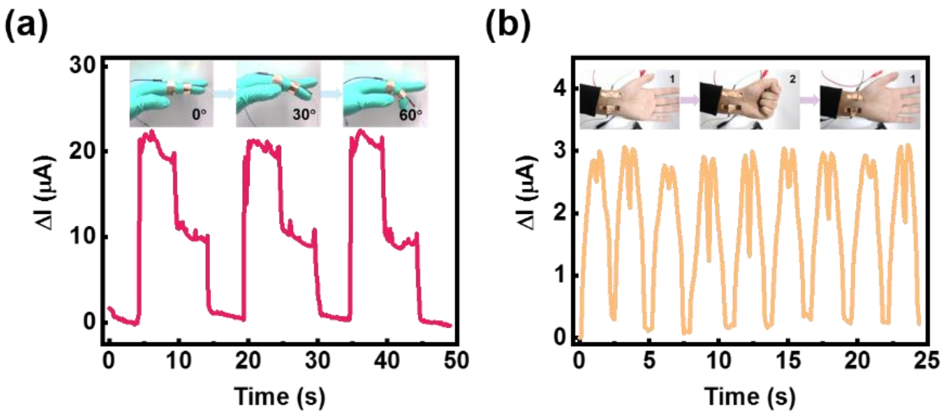


Figure S12. (a) The sensing curve of the detection finger was maintained at 0°, 30°, and 60°. Each of these states was maintained for 5 seconds. (b) Sensing curve for wrist clenched and relaxed.
